# Hypercalcemia-Associated Hodgkin's Lymphoma Presenting as Normal Pressure Hydrocephalus: A Case Report

**DOI:** 10.7759/cureus.71471

**Published:** 2024-10-14

**Authors:** Sean Lief, Ali Z Ansari, Samer Beauti, Axel B Lichtenberg, Srihita Patibandla, Azouba Gulraiz, Rahul R Tirumalareddy, Mashaal Khan

**Affiliations:** 1 Department of Internal Medicine, William Carey University College of Osteopathic Medicine, Hattiesburg, USA; 2 Department of Pathology, William Carey University College of Osteopathic Medicine, Hattiesburg, USA; 3 Department of Orthopedic Surgery, William Carey University College of Osteopathic Medicine, Hattiesburg, USA; 4 Department of Internal Medicine, Trinity Health Grand Rapids, Grand Rapids, USA; 5 Department of Internal Medicine, Poplar Bluff Regional Medical Center, Poplar Bluff, USA; 6 Department of Internal Medicine, The University of Tennessee Health Science Center, Memphis, USA

**Keywords:** back pain, brentuximab vedotin, central disc protrusion, hodgkin's lymphoma, hypercalcemia, lumbar spinal canal stenosis, metastatic disease, normal pressure hydrocephalus, reed-sternberg cells, thoracentesis

## Abstract

Hodgkin's lymphoma is a malignancy of the lymphatic system that can rarely present with advanced-stage features such as spinal cord involvement and hypercalcemia. We present the case of a 63-year-old Caucasian male with advanced Hodgkin's lymphoma, presenting with hypercalcemia and symptoms resembling normal pressure hydrocephalus. The patient exhibited gait impairment, altered mental status, and urinary incontinence, forming the classic triad seen in normal pressure hydrocephalus. Laboratory findings revealed leukocytosis, anemia, hypercalcemia, and acute kidney injury. Imaging showed extensive lymphadenopathy, a right adrenal mass, and bone marrow abnormalities suggestive of disseminated malignancy, leading to the decision not to biopsy the adrenal mass due to the strong suspicion of widespread metastatic disease. A biopsy of a mediastinal lymph node confirmed the diagnosis of Hodgkin's lymphoma, with hypercalcemia attributed to paraneoplastic syndrome. The patient's treatment included managing hypercalcemia and administering chemotherapy with adriamycin, bleomycin, vinblastine, and dacarbazine. This case highlights the importance of considering Hodgkin's lymphoma in patients with atypical presentations and the need for comprehensive diagnostic evaluation, including imaging and laboratory tests. While hypercalcemia in Hodgkin's lymphoma is associated with a poor prognosis, newer therapies like brentuximab vedotin offer promising treatment options for frail patients.

## Introduction

Hodgkin's lymphoma is a rare malignancy of the lymphatic system. It is one of the three main types of liquid tumors, also known as non-solid tumors or blood cancers, alongside leukemia and multiple myeloma [1]. Lymphomas are categorized into two main types: Hodgkin's lymphoma and non-Hodgkin's lymphoma. Hodgkin's lymphoma constitutes approximately 10% of all diagnosed lymphomas, making it less common than non-Hodgkin's lymphoma [2]. It typically exhibits a bimodal age distribution, with the first peak occurring in young adults and the second in older adults, usually in late middle age or beyond [2-4]. Notably, lymphoma is the most prevalent cancer in individuals aged 15-19, with about two-thirds of these cases being Hodgkin's lymphoma [2]. The diagnosis is confirmed through biopsy, which reveals the characteristic Reed-Sternberg cells.

Spinal metastasis in Hodgkin's lymphoma is an uncommon manifestation, occurring in approximately 5.8% of patients [5]. When present, it often indicates advanced disease with widespread dissemination. Given its rarity, symptoms such as impaired ambulation, urinary incontinence, and confusion in patients with Hodgkin's lymphoma may initially be misinterpreted as normal pressure hydrocephalus. Normal pressure hydrocephalus is characterized by a triad of dementia, gait disturbances, and urinary incontinence [6]. This atypical presentation may be better explained by the combination of spinal cord lesions, which induce neuromuscular weakness affecting gait and urinary control, along with a neurocognitive disorder secondary to hypercalcemia.

## Case presentation

A 63-year-old Caucasian male presented to the emergency department (ED) with symptoms of increased weakness, an inability to walk, and altered mental status. Over the past 72 hours, he experienced worsening confusion, urinary incontinence, and decreased appetite, accompanied by nausea. His medical history includes hypertension, type 2 diabetes mellitus, and chronic back pain. He also has a surgical history of left rotator cuff repair and lumbar spinal decompression surgery. The patient's sister and daughter provided his medical history, noting that he had not been himself for several weeks, exhibiting significant weakness, lethargy, and an inability to care for himself. His daughter also reported substantial weight loss and excessive sleep. He had recently moved to Mississippi from Ohio approximately six months ago, and his daughter observed a decline in his health since the move. The patient is a current cigarette smoker with a history of intermittent smoking throughout his adult life. Before retiring, he worked in the car detailing industry. The family denied any symptoms of cough, congestion, fever, chills, changes in bowel habits, abdominal pain, or syncopal episodes.

Three days prior to admission, the patient was evaluated by his primary care physician (PCP), who identified abnormal laboratory results; however, the specifics were not available to the family. He was scheduled for a computed tomography (CT) scan and magnetic resonance imaging (MRI), but these were not completed before his admission. Upon admission, laboratory findings were significant for leukocytosis, anemia, thrombocytosis, elevated blood urea nitrogen (BUN), and acute kidney injury with a creatinine level of 2.4 mg/dL (Table 1). Additional findings included hyperglycemia, hypercalcemia with a corrected calcium level of 11.8 mg/dL, hypoalbuminemia, elevated alkaline phosphatase (ALP), elevated aspartate aminotransferase (AST), elevated total protein, and hypermagnesemia.

**Table 1:** Laboratory findings at the time of admission.

Test	Observed Value	Reference Range
White blood cells	19.3 x 10^3^/µL	4.0-11.0 x 10^3^/µL
Red blood cells	2.8 x 10^6^/µL	4.0-5.0 x 10^6^/µL
Hemoglobin	7.5 g/dL	12.1-15.1 g/dL
Hematocrit	23%	42%-52%
Mean corpuscular hemoglobin	27 pg/cell	27-31 pg/cell
Mean corpuscular hemoglobin concentration	33 g/dL	33-36 g/dL
Mean corpuscular volume	83 fL	80–100 fL
Platelet count	527 x 10^9^/L	150-450 x 10^9^/L
Mean platelet volume	8 fL	8-12 fL
Red blood cell distribution width	17%	12%-15%
Sodium	135 mmol/L	135-147 mmol/L
Potassium	4.3 mmol/L	3.5-5.0 mmol/L
Chloride	99 mmol/L	96-106 mmol/L
Carbon dioxide	25 mmol/L	23-29 mmol/L
Blood urea nitrogen	9.3 mmol/L	2.1-8.5 mmol/L
Creatinine	2.4 mg/dL	0.7-1.3 mg/dL
Glucose	169 mg/dL	70-100 mg/dL
Calcium	10.3 mg/dL	8.5-10.2 mg/dL
Albumin	2.1 g/dL	3.5-5.5 g/dL
Alkaline phosphatase	225 U/L	44-147 U/L
Alanine aminotransferase	32 U/L	7-56 U/L
Aspartate aminotransferase	63 U/L	5-40 U/L
Total bilirubin	0.9 mg/dL	0.3-1.0 mg/dL
Total protein	8.8 g/dL	6.0-8.3 g/dL
Globulin	2.9 g/dL	2.0-3.5 g/dL
Magnesium	3.0 mEq/L	1.3-2.1 mEq/L
Corrected calcium	11.8 mg/dL	8.5-10.2 mg/dL

Given the patient's initial presentation, which included urinary incontinence, gait disturbances, and cognitive decline, there was clinical suspicion of normal pressure hydrocephalus. An MRI of the brain was performed to evaluate for normal pressure hydrocephalus; however, the imaging did not reveal any abnormalities. Additionally, the Evans index, a measurement used in the assessment of normal pressure hydrocephalus, was calculated to be 0.12, well below the diagnostic threshold of 0.3. Consequently, normal pressure hydrocephalus was ruled out as a potential diagnosis.

**Figure 1:**
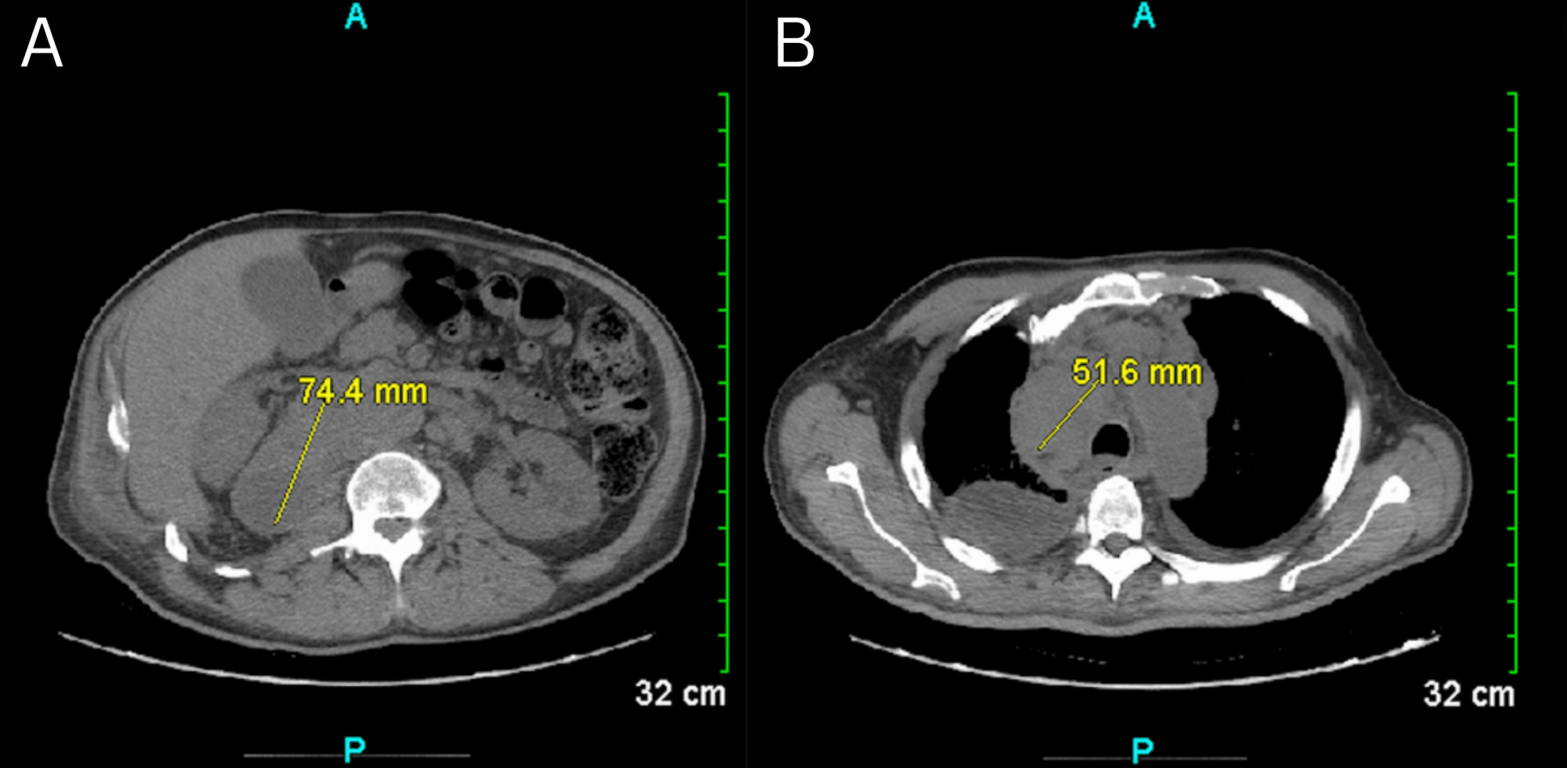
The CT scan shows a mass within the right adrenal gland that extends inferiorly and medially, displacing the right kidney, with a measurement of at least 7 cm (A). There is abnormal soft tissue in the right paratracheal region, consistent with lymphadenopathy, measuring at least 5 cm (B). Additionally, extensive atelectasis in the right lung obscures the right hilum, and a small left pleural effusion is also observed. CT: computed tomography.

A CT scan of the chest revealed a large right paratracheal lymph node and an adrenal mass, both suspicious for neoplasm, along with a large right pleural effusion associated with extensive atelectasis and a small left pleural effusion (Figure 1). A large-volume thoracentesis was performed, during which 1,060 mL of bloody fluid was aspirated from the right chest. Pleural fluid analysis was exudative, with a lactate dehydrogenase (LDH) level of 296 U/L, a protein concentration of 5.1 g/dL, and a glucose level of 117 mg/dL. The cell count revealed 62,000 red blood cells and 68% lymphocytes.

**Figure 2:**
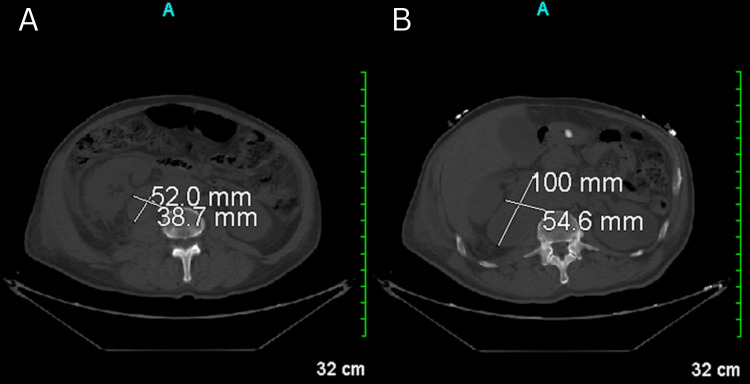
The CT scan reveals upper abdominal lymphadenopathy and extensive retroperitoneal lymphadenopathy. An index lymph node mass posterior to the right kidney and lateral to the inferior vena cava measures 5.2 x 3.9 cm (A), while another index lymph node mass in the upper abdomen measures 10 x 5.5 cm (B). Gallstones are present. Additionally, a small amount of ascites is observed. CT: computed tomography.

**Figure 3:**
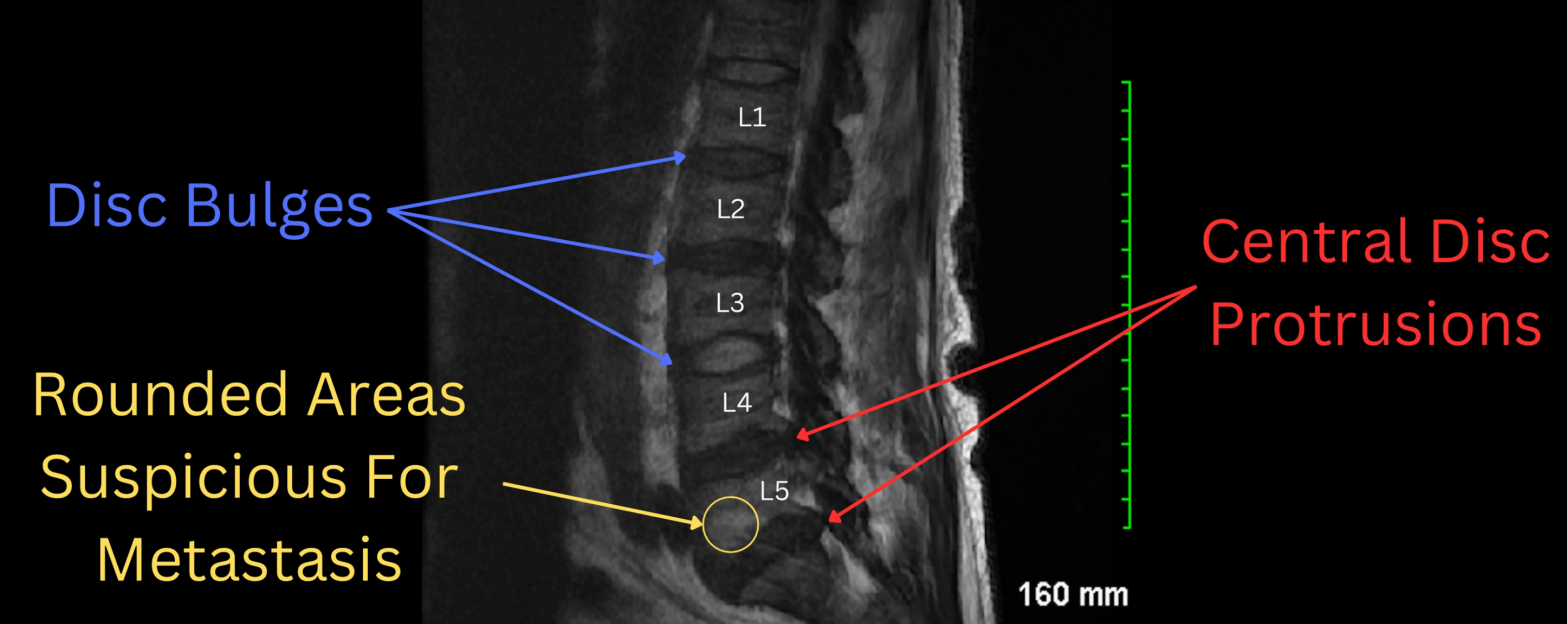
The MRI scan reveals the following findings at various lumbar levels. At L1-L2, there is a broad-based disc bulge (blue arrow) and mild facet changes. At L2-L3 and L3-L4, there are broad-based disc bulges (blue arrows) with moderate facet changes. At L4-L5, a large central disc protrusion (red arrow) is noted, accompanied by facet changes, and there is narrowing of the spinal canal. At L5-S1, a central disc protrusion (red arrow) with facet changes is observed. Additionally, the marrow signal is diffusely abnormal, with multiple rounded areas of abnormal signal (yellow circle and yellow arrow) that raise suspicion for possible metastatic disease. CT: computed tomography.

A CT scan of the abdomen revealed abdominal lymphadenopathy, extensive retroperitoneal lymphadenopathy, and a right adrenal mass (Figure 2). Further imaging with an MRI of the lumbar spine showed bone marrow signal abnormalities suspicious for metastatic disease, along with multilevel degenerative changes (Figure 3). These findings, combined with the lymphadenopathy and adrenal mass, suggested a disseminated malignancy. Given the overall presentation, including systemic symptoms and neurological deficits, a biopsy of the right adrenal mass was not performed. The clinical team opted against biopsy due to the strong suspicion of widespread metastatic disease, as indicated by the imaging findings.

**Figure 4:**
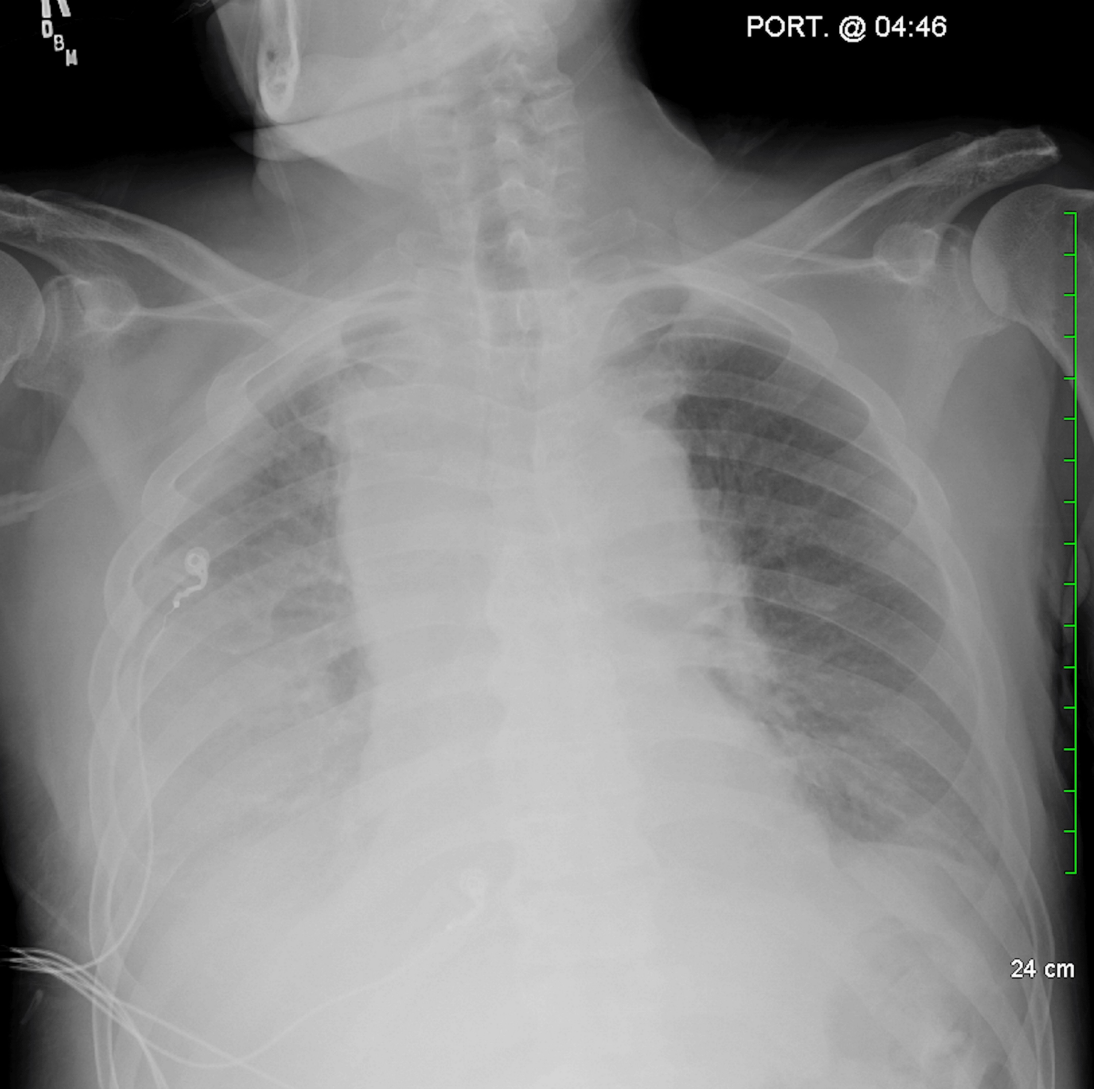
The chest X-ray shows a right pleural effusion, which appears to have increased in size compared to the previous CT scan. There is also mediastinal widening with soft tissue density, consistent with the patient’s known history of lymphadenopathy. CT: computed tomography.

Two days after admission, the patient developed new-onset shortness of breath. Although a CT scan had already identified pleural effusions, a chest X-ray was ordered to assess the patient’s acute respiratory status and evaluate for any changes in the effusions or new complications, such as worsening effusions or pneumonia, that could have developed since the CT scan. The X-ray confirmed persistent right pleural effusion and mediastinal lymphadenopathy (Figure 4). Notably, the right pleural effusion appeared to have increased in size compared to the initial CT scan findings, indicating progression despite the previous thoracentesis. Given the presence of pleural effusion and respiratory symptoms, management included supportive care with supplemental oxygen and careful monitoring of respiratory status. No further thoracentesis was performed at this stage, as the patient’s symptoms were stable following the initial drainage.

**Figure 5:**
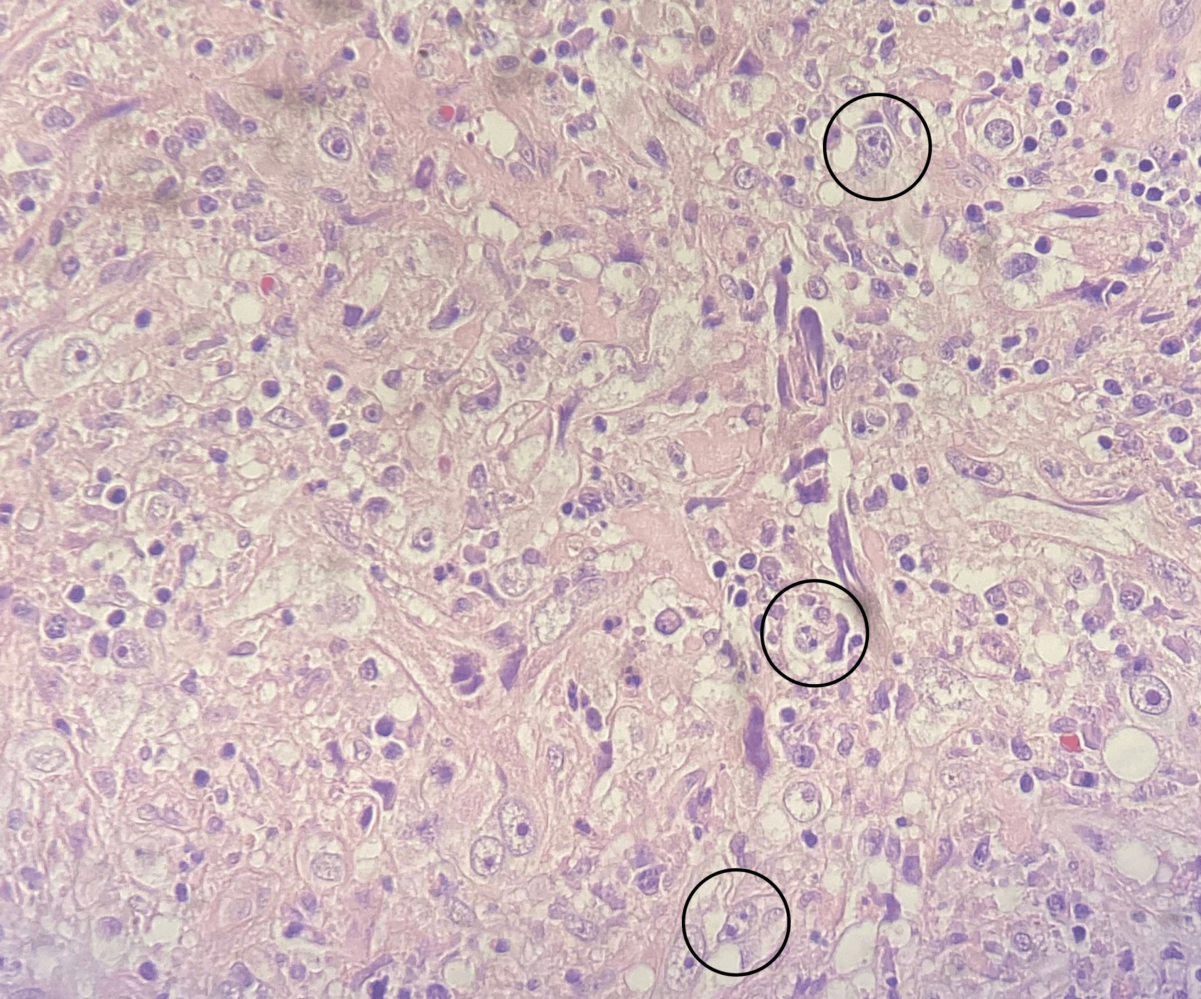
Biopsy of a mediastinal lymph node revealing the presence of multiple Reed-Sternberg cells (black circles) with complete lymph node effacement.

A biopsy of a mediastinal lymph node revealed the presence of Reed-Sternberg cells, consistent with Hodgkin's lymphoma (Figure 5). Immunohistochemistry staining was performed, with CD3, CD20, and CD45 being negative, while CD15, CD30, and PAX5 were positive, further confirming the diagnosis of Hodgkin's lymphoma. The patient’s hypercalcemia was determined to be paraneoplastic in nature, likely related to increased production of bioactive vitamin D by the lymphoma cells. Further staging workup indicated advanced disease with extranodal involvement, including possible spinal metastasis contributing to the patient’s symptoms of gait disturbance and urinary incontinence.

The patient’s treatment plan included the initial management of hypercalcemia with intravenous fluids and pamidronate, along with monitoring and managing acute kidney injury. Bisphosphonates were administered within the first 24 hours, with the dosage adjusted based on renal function. Oncologic treatment was initiated with a chemotherapy regimen of adriamycin, bleomycin, vinblastine, and dacarbazine. Given the patient’s advanced age and frailty, consideration was given to using brentuximab vedotin, a targeted antibody-drug conjugate, as an alternative or adjunct to standard chemotherapy. Symptomatic management included palliative care for symptom control and support, with consideration of hospice care due to the advanced stage of the disease and poor prognostic factors, including hypercalcemia and spinal metastasis.

The patient’s condition was closely monitored throughout the treatment process. Despite aggressive therapy, the prognosis remained uncertain due to the advanced stage of the disease and the presence of hypercalcemia, which is associated with a poor prognosis in Hodgkin’s lymphoma. The patient and his family were counseled on the likely progression of the disease and the potential need for hospice care as the condition advanced.

## Discussion

Hodgkin’s lymphoma presenting with hypercalcemia, along with difficulty ambulating, urinary incontinence, and confusion, creates a rare and misleading clinical picture that may initially suggest normal pressure hydrocephalus, especially when symptoms progress over a six-month period [6]. While the literature on Hodgkin’s lymphoma presenting with hypercalcemia is limited, reported incidence rates range from 0.9% to 5.4% in patients with classical Hodgkin’s lymphoma [7]. This unusual presentation can be attributed to a combination of advanced and uncommon features of Hodgkin’s lymphoma, such as spinal cord metastasis, along with the atypical electrolyte imbalance caused by hypercalcemia [5,7]. This highlights the need for careful consideration of malignancy in differential diagnoses when faced with such clinical presentations.

Hodgkin’s lymphoma most commonly presents in the mediastinal region or cervical lymph nodes. While the disease often manifests with vague or nonspecific symptoms, approximately 35% of patients exhibit systemic symptoms known as B-symptoms, including chills, fever, night sweats, and weight loss [2-4]. In contrast, our patient experienced chronic back pain, unexplained weight loss, and weakness but did not present with the typical B-symptoms of chills, fever, or night sweats. Notably, the patient exhibited significant altered mental status at onset, to the extent that a family member had to provide a detailed medical history. This emphasizes the importance of considering various potential causes for symptoms such as urinary incontinence, including neurological conditions like diabetic neuropathy or motor neuron lesions, as well as functional incontinence resulting from cognitive and mobility impairments [8].

Hypercalcemia in malignancy often indicates advanced disease and can lead to significant neurological symptoms, as observed in our patient. This condition may suggest bone metastasis, where osteoclast activation results in increased bone resorption and elevated serum calcium levels. In Hodgkin's lymphoma, hypercalcemia is typically associated with the production of high levels of bioactive vitamin D (calcitriol) by tumor cells [9]. While the differential diagnosis for hypercalcemia is broad, it can often be narrowed by assessing parathyroid hormone (PTH) levels. Elevated PTH may indicate a parathyroid adenoma, whereas low PTH is more suggestive of malignancy-related hypercalcemia [10]. Unfortunately, hypercalcemia due to malignancy is associated with a poor prognosis, often resulting in a significantly reduced mean survival time [9,10].

For the evaluation of lymphomas, positron emission tomography (PET) is recommended for diagnosing fluorodeoxyglucose (FDG)-avid lymphomas, such as Hodgkin’s lymphoma. If the lymphoma is less FDG-avid, CT is preferred. When bone marrow involvement is suspected in Hodgkin’s lymphoma, bone marrow biopsy has traditionally been the gold standard; however, PET-CT’s high sensitivity often renders bone marrow biopsy unnecessary if a PET-CT has already been performed [11]. Awareness of the common symptoms associated with spinal cord metastasis, including back pain, is essential for accurate diagnosis [12]. Prognostic factors such as anemia, which was present in our patient, can indicate a poor prognosis. Treatment for Hodgkin’s lymphoma typically involves a combination of chemotherapy and radiotherapy. The standard chemotherapy regimen, adriamycin, bleomycin, vinblastine, and dacarbazine, has significantly improved survival rates, with a five-year survival rate now around 89.8% for patients aged 20-64, compared to under 10% before this regimen became standard [2]. Novel therapies, such as brentuximab vedotin, a CD30-targeted antibody-drug conjugate, have shown promise, particularly in elderly or frail patients who may not tolerate traditional regimens due to systemic toxicity [1,2].

## Conclusions

Advanced Hodgkin's lymphoma with hypercalcemia and spinal cord involvement can present with symptoms similar to the triad seen in normal pressure hydrocephalus. This case highlights the importance of considering malignancy in patients presenting with failure to thrive and weakness that may mimic neurological conditions. Although hypercalcemia is associated with a poorer prognosis, Hodgkin's lymphoma remains treatable at all stages with chemotherapy and radiotherapy. For frail patients unable to tolerate traditional chemotherapy, newer therapies with lower systemic toxicity, such as brentuximab vedotin, offer promising treatment options. This development represents a hopeful direction in the treatment of Hodgkin's lymphoma, potentially improving outcomes for affected patients. Given the significant prognostic implications of hypercalcemia in malignancy, further research into specific treatments targeting this electrolyte imbalance is warranted.
